# Polycystic ovary syndrome as a plausible evolutionary outcome of metabolic adaptation

**DOI:** 10.1186/s12958-021-00878-y

**Published:** 2022-01-10

**Authors:** Daniel A. Dumesic, Vasantha Padmanabhan, Gregorio D. Chazenbalk, David H. Abbott

**Affiliations:** 1 Department of Obstetrics and Gynecology, David Geffen School of Medicine at UCLA, 10833 Le Conte Ave, Room 22-178 CHS, Los Angeles, CA 90095 USA; 2 Department of Pediatrics, University of Michigan, Ann Arbor, MI 48109 USA; 3 Department of Obstetrics and Gynecology, University of Wisconsin and Wisconsin National Primate Research Center, 1223 Capitol Court, Madison, WI 53715 USA

**Keywords:** Polycystic ovary syndrome, Hyperandrogenism, Insulin resistance, Adipocyte, Adipose stem cells, Developmental programming, Nonhuman primates, Sheep, Body fat distribution, Metabolic adaptation

## Abstract

As a common endocrinopathy of reproductive-aged women, polycystic ovary syndrome (PCOS) is characterized by hyperandrogenism, oligo-anovulation and polycystic ovarian morphology. It is linked with insulin resistance through preferential abdominal fat accumulation that is worsened by obesity. Over the past two millennia, menstrual irregularity, male-type habitus and sub-infertility have been described in women and confirm that these clinical features of PCOS were common in antiquity. Recent findings in normal-weight hyperandrogenic PCOS women show that exaggerated lipid accumulation by subcutaneous (SC) abdominal stem cells during development to adipocytes in vitro occurs in combination with reduced insulin sensitivity and preferential accumulation of highly-lipolytic intra-abdominal fat in vivo. This PCOS phenotype may be an evolutionary metabolic adaptation to balance energy storage with glucose availability and fatty acid oxidation for optimal energy use during reproduction. This review integrates fundamental endocrine-metabolic changes in healthy, normal-weight PCOS women with similar PCOS-like traits present in animal models in which tissue differentiation is completed during fetal life as in humans to support the evolutionary concept that PCOS has common ancestral and developmental origins.

## Background

Polycystic ovary syndrome (PCOS) is characterized by ovarian hyperandrogenism from altered hypothalamic-pituitary-ovarian function in combination with hyperinsulinemia from insulin resistance. With a prevalence of 6–20% in the general population, depending upon the definition of PCOS [1], its clinical manifestations of hirsutism, oligo-anovulation and polycystic ovarian morphology (PCOM) accompany glucose intolerance, dyslipidemia and preferential abdominal fat accumulation worsened by obesity [2]. These clinical manifestations of PCOS determine risks of subfertility, diabetes, metabolic syndrome and/or cardiovascular disease. Almost one-half of women with PCOS in the United States have metabolic syndrome, which is higher in prevalence than that of age-matched normal women in this country [1, 3] and of women with PCOS in other countries where obesity is less prevalent [4].

As a heritable syndrome with a polygenic origin, large genome-wide association studies (GWAS) have identified several PCOS susceptible loci in candidate genes involving insulin action, androgen biosynthesis and gonadal function [1]. These PCOS susceptible loci alone, however, have yet to explain the majority of PCOS phenotypic expression [5]. Rather, heritability of PCOS may involve one or more PCOS candidate genes interacting with environmental factors to modify target tissue phenotype through epigenetic events [6, 7], beginning before birth when an altered maternal endocrine-metabolic environment modifies fetal genetic susceptibility to PCOS, and continuing after birth into adulthood [4].

This review examines how the endocrine-metabolic characteristics of women with PCOS originally favored survival of humans in ancient times of food deprivation, but now predispose to endocrine-reproductive dysfunction in today’s obesogenic environment. It integrates clinical characteristics of PCOS women with similar PCOS-like traits present in prenatally testosterone-treated monkeys and sheep to provide evidence of developmental programming in PCOS, given that tissue differentiation in these species, as in humans, occurs during fetal life.

### Clinical variables

Several variables affect the endocrine-metabolic characteristics of women with PCOS. As one such variable, different PCOS phenotypes by Rotterdam criteria vary in their degree of reproductive and metabolic dysfunction [8]. Women with National Institutes of Health (NIH)-defined PCOS (i.e., hyperandrogenism with oligo-anovulation with or without PCOM) are at greatest risk of developing menstrual irregularity, anovulatory infertility, type 2 diabetes mellitus and metabolic syndrome, as defined by increased abdominal (android) obesity, hyperglycemia, dyslipidemia and hypertension. Ovulatory women with PCOS (i.e., hyperandrogenism and PCOM) have a lower body mass index (BMI) and milder hyperinsulinemia and hyperandrogenism, which lower the risks of developing reproductive and metabolic abnormalities, while women with non-androgenic PCOS (oligo-anovulation and PCOM) have the least metabolic risk [1].

In addition, obesity coexists with abnormal insulin action in most women with PCOS [9–11]. Although not an intrinsic defect of PCOS, obesity can interact with hyperandrogenism to worsen PCOS phenotypes [1–3, 12–14] and impair insulin sensitivity [2, 15, 16]. In this regard, women with PCOS within a referral population have a more severe phenotype, including greater hyperandrogenism, higher BMI and increased risk for metabolic dysfunction, than PCOS women within an unselected background population [17, 18]. Age is another positive predictor of insulin resistance in adipose tissue [19]. These variables that adversely affect endocrine-metabolic function in PCOS were avoided in our studies by investigating healthy, normal-weight PCOS women by NIH criteria who were recruited from the general population to study a mild PCOS phenotype [17, 20] and who also were age- and BMI-balanced to controls to eliminate the effects of age and obesity on metabolic outcomes, including insulin sensitivity [15, 16, 19, 21].

In the context of metabolic function, women with NIH-defined PCOS have two distinct PCOS subtypes with different genetic heterogeneity. A reproductive endocrine subtype (23% of cases) is characterized by higher luteinizing hormone (LH) and sex hormone binding globulin (SHBG) levels with relatively low BMI and insulin levels, while a metabolic subtype (37% of cases) is characterized by higher BMI, glucose and insulin levels, with lower SHBG and LH levels. These PCOS subtypes may differ in their developmental origins [22], with their heritability variably interacting with risk-increasing environmental factors, including maternal obesity and gestational diabetes, to fully explain its prevalence. Such genetic-environmental interactions likely begin before birth, when an altered maternal-placental-fetal environment generates epigenetic modifications in fetal genetic susceptibility to PCOS that continue after birth into adulthood, with metabolic adaptations that enhance fat storage but predispose to lipotoxicity [23].

### Insulin resistance

Most women with PCOS have some degree of insulin resistance due to perturbed insulin receptor/post receptor signaling, altered adipokine secretion and abnormal steroid metabolism [2] in combination with increased abdominal fat over a wide BMI range [1, 24, 25]. Clinically, insulin sensitivity (Si) and insulin resistance can be quantified by frequently sampled intravenous glucose tolerance testing (FSIVGTT) and/or Homeostatic Model Assessment for Insulin Resistance (HOMA-IR), respectively. Healthy, normal-weight PCOS women by NIH criteria have Si and HOMA-IR values within low-normal and high-normal and ranges, respectively [26]. By total body dual-energy x-ray absorptiometry, they also exhibit preferential accumulation of abdominal fat, called android fat, which positively correlates with circulating androgen and fasting insulin levels, and remains related to serum androgen levels adjusting for serum insulin levels [24].

### Intra-abdominal adipose

In women with NIH-defined PCOS, preferential abdominal fat accumulation [2, 27] promotes insulin resistance through increased intra-abdominal (visceral) fat mass, and worsens with weight gain as a risk factor for metabolic disease [28–30]. Such preferential abdominal fat accumulation in hyperandrogenic PCOS women underlies insulin resistance over a wide BMI range [25], and also occurs in healthy normal-weight PCOS women by NIH criteria in combination with adipose insulin resistance (adipose-IR)*,* defined by the product of fasting circulating free fatty acid (FFA) and insulin levels [19, 24, 26]. In these normal-weight PCOS women, moreover, an increase in intra-abdominal fat positively correlates with serum androgen concentrations and fasting circulating levels of insulin, triglyceride (TG), as well as non-high-density lipoprotein (non-HDL) cholesterol [24].

Intra-abdominal adipose in humans is highly lipolytic and resists androgen inhibition of catecholamine-induced lipolysis (lipid breakdown) despite expressing androgen receptors [31]. Instead, intra-abdominal adipose of nonobese PCOS women shows exaggerated catecholamine-induced lipolysis despite normal insulin suppression of lipolysis [32, 33]. Consequently, an increase in intra-abdominal fat in normal-weight PCOS women likely enhances FFA delivery to the liver and muscle for energy storage, but worsens insulin resistance if increased FFA availability exceeds the capacity of these tissues to oxidize fat or convert diacylglycerols to triacylglycerols [32–34].

### Subcutaneous abdominal adipose

Subcutaneous (SC) abdominal adipose normally protects against insulin resistance through a balance between lipogenesis (lipid formation) and lipolysis (lipid breakdown) in mature adipocytes combined with new adipocyte formation (i.e., adipogenesis), whereby adipose stem cells (ASCs) initially undergo commitment to preadipocytes and then differentiate into newly-formed adipocytes [35–37]. Subcutaneous adipose can thus increase its fat storage capacity through both enlargement of mature adipocytes (i.e., hypertrophy) and development of new adipocytes (i.e., hyperplasia) to buffer fatty acid influx when energy intake exceeds energy expenditure [38, 39].

Within SC adipose, androgen normally inhibits early-stage adipogenesis, diminishes insulin-stimulated glucose uptake and impairs catecholamine-stimulated lipolysis through reduced β_2_-adrenergic receptor and hormone-sensitive lipase (HSL) protein expression [31, 32, 35, 40]. As a result, SC abdominal adipose of PCOS women shows diminished insulin-mediated glucose uptake, reduced glucose transporter type 4 (GLUT-4) expression [41] and catecholamine lipolytic resistance from diminished protein levels of β_2_-adrenergic receptor, HSL and protein kinase A regulatory-IIβ component (PKA-RegIIβ) [42, 43]. As a biomarker of lipolysis, serum glycerol levels are decreased in normal-weight PCOS women with normal insulin sensitivity [42], yet increased in overweight PCOS women [44], likely because androgen-induced catecholamine lipolytic resistance in normal-weight PCOS women [43] is antagonized by impaired insulin suppression of lipolysis in overweight PCOS women [44].

Consequently, adipose-IR is often increased in healthy normal-weight women with NIH-defined PCOS compared to age- and BMI-matched normal women [26]. Moreover, adipose-IR in these women positively correlates with serum androgen and fasting TG levels, and negatively correlates with Si values [26]. These endocrine-metabolic relationships are further modified by local intracellular aldo-ketoreductase type 1C3 (AKR1C3) activity, an aldo-ketoreductase enzyme that generates testosterone (T) from androstenedione (A4) and exists predominantly in SC rather than intra-abdominal adipose [45, 46]. In overweight/obese PCOS women, increased AKR1C3-mediated T generation from A4 in SC abdominal adipose enhances lipid storage through increased lipogenesis and decreased lipolysis [47]. Similarly, in normal-weight women with NIH-defined PCOS, an increased serum total T/A4 ratio, as a marker of enhanced SC adipose AKR1C3 activity, negatively correlates with fasting serum TG levels, adjusting statistically for serum free T as a possible confounding variable [48]. This inverse relationship of serum total T/A4 ratio with fasting serum TG level in normal-weight, NIH-defined PCOS women may reduce TG turnover to counter-balance androgen inhibition of insulin-stimulated glucose uptake in SC abdominal adipocytes [40].

### SC abdominal stem cells

The dynamic process of adipocyte development begins in early life [49]. Subcutaneous abdominal ASCs from normal-weight PCOS by NIH criteria compared to age−/BMI matched normal healthy women exhibit inherently altered gene expression of adipogenic/angiogenic functions involving androgen-insulin interactions through transforming growth factor (TGF)-β1 signaling [26]. When SC abdominal ASCs from normal-weight PCOS women are cultured in vitro (without exogenous androgen), exaggerated commitment of ASC to preadipocytes via zinc-finger protein 423 (ZFP423) expression negatively correlates with fasting circulating glucose levels [50]. Subsequently, accelerated lipid accumulation in newly-formed PCOS adipocytes during adipocyte maturation in vitro positively correlates with hyperandrogenemia and predicts both reduced serum FFA levels and improved systemic insulin sensitivity in vivo [50, 51]. In some SC abdominal ASCs from normal-weight PCOS, overexpression of peroxisome proliferator-activated receptor *γ* (*PPARγ*) and CCAAT enhancer binding protein *a (CEBPa*) during adipocyte maturation in vitro accompanies altered dynamic chromatin remodeling, with enrichment of binding motifs for transcription factors of the activator protein-1 (AP-1) subfamily that govern adipocyte differentiation [23]. These findings suggest that triacylglycerol synthesis, lipid oxidation, free fatty acid beta-oxidation and oxidative phosphorylation may be reprogrammed in these cells to promote greater fat storage [50]. Similar studies of SC abdominal ASC gene expression and function in overweight/obese PCOS by NIH criteria have not yet been performed.

Linked with this exaggerated ZFP423-induced ASC commitment to preadipocytes in vitro is a greater formation of small SC abdominal adipocytes in normal-weight PCOS women [24, 26, 50]. A similar population of small SC abdominal adipocytes in other individuals [49, 52, 53] protects against insulin resistance through enhanced ASC commitment to preadipocyte differentiation and *ZFP423* upregulation due to epigenetic changes in its promoter region [54]. An increased proportion of small SC abdominal adipocytes occurs in PCOS-like prenatally-T treated adult rhesus monkeys with increased visceral adiposity and insulin resistance [5, 55]. It also occurs in prenatally-T treated sheep with insulin resistance [56], perhaps as a compensatory adaptation to an altered intrauterine metabolic environment apart from extant androgen since enhancement of the small adipocyte population is not reversed with flutamide cotreatment [57, 58].

Finally, overexpression of *PPARγ* and *CEBPa* in some PCOS SC abdominal stem cells accompanies upregulation of *AKR1C3* during adipocyte maturation in vitro. These findings correspond with an inverse relationship of serum total T/A4 ratio with serum TG level in normal-weight PCOS women [48], and suggest that reduced TG turnover in SC adipose of these individuals favors insulin sensitivity [59, 60]. AKR1C3 gene expression and activity are normally greater in preadipocytes and adipose of gluteal compared to omental fat, with gluteal fat favoring androgen activation (i.e., AKR1C3), and omental cells favoring androgen inactivation (i.e., aldo-ketoreductase type 1C2 [AKR1C2]) [46]. These differential actions of AKR1C3-mediated androgen activation by fat depot, combined with hyperandrogenemia and preferential intra-abdominal fat accumulation, likely influence body fat distribution and function in normal-weight PCOS women through a programmed mechanism to balance glucose-insulin homeostasis with fat accretion [24, 50].

### Lipotoxicity

**Fig. 1:**
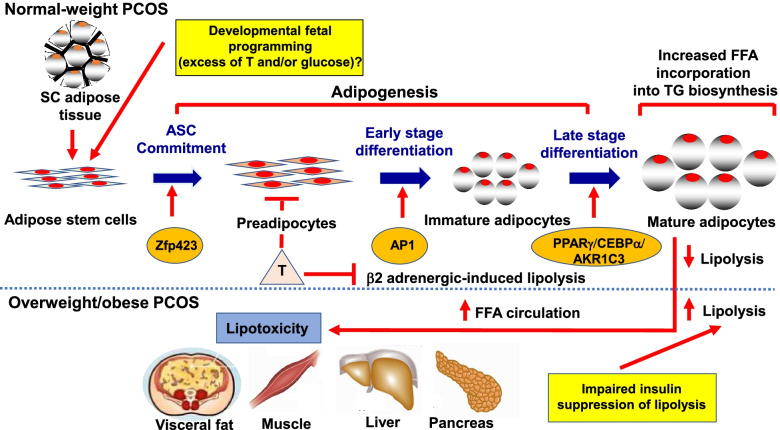
Altered molecular pathways of subcutaneous (SC) abdominal adipogenesis in polycystic ovary syndrome (PCOS) as a risk factor for lipotoxicity. In normal-weight PCOS women, exaggerated adipose stem cell (ASC) development to adipocytes occurs via androgen-independent mechanisms [23, 50]. Simultaneously, androgen excess inhibits early-stage adipogenesis, diminishes insulin-stimulated glucose uptake, promotes lipid storage and impairs catecholamine-stimulated lipolysis [31, 32, 35, 40, 47], favoring abdominal fat deposition and increased energy availability through hyperandrogenism and insulin resistance, respectively. These same traits are worsened in overweight/obese PCOS women who have greater preferential abdominal fat accumulation, hyperandrogenism, and systemic insulin resistance [2], along with impaired insulin suppression of lipolysis [2, 44], promoting ectopic lipid deposition and lipotoxicity [1]

Several endocrine-metabolic characteristics of PCOS women worsen with increased adiposity. Overweight/obese compared to normal-weight women with PCOS exhibit greater preferential abdominal fat accumulation, hyperandrogenism and insulin resistance [2] accompanied by increased serum glycerol levels from impaired insulin suppression of lipolysis [44]. Their enlarged SC abdominal mature adipocytes are also more pre-disposed to a pro-inflammatory lipid depot environment than the increased number of smaller SC abdominal adipocytes present in normal-weight women with PCOS [24, 44]. Given androgen inhibition of early-stage SC abdominal adipogenesis [35], overweight/obese women with PCOS are more likely than normal-weight PCOS women to have an impaired ability to properly store fatty acid influx in SC fat as energy intake exceeds energy expenditure [38, 39], promoting ectopic lipid deposition in non-adipose tissue [1] (Fig. 1).

Lipotoxicity refers to the ectopic lipid accumulation in non-adipose tissue where it induces oxidative/endoplasmic reticulum stress tightly linked with insulin resistance and inflammation [61]. Overweight/obese women with PCOS are at increased risk of developing lipotoxicity due to excess FFA uptake into non-adipose cells, including the muscle, liver, pancreas and ovary, which is exacerbated by increased intra-abdominal fat with high lipolytic activity [32, 33, 62–65]. Consequently, excess fatty acid influx into skeletal muscle and liver promotes diacylglycerol-induced insulin resistance, which impairs insulin signaling via increased insulin receptor serine phosphorylation, and worsens with disrupted mitochondrial oxidative phosphorylation [34, 66].

### Developmental programming: dual hits from maternal-fetal endocrine-metabolic dysfunction

Through an evolutionary perspective, the high worldwide prevalence of PCOS in today’s environment and its negative impact on reproduction should have disappeared over millennia unless a beneficial effect favored both survival and reproduction [67]. There is little genetic evidence of strong selection against transmission of PCOS risk genes across multiple generations [68]. Rather, ancestral traits may have originally favored PCOS in hunter-gatherers of the late Pleistocene, or perhaps in more ancient human populations, when scarcity of food in pregnancy programmed in the fetus enhanced adipogenesis for greater fat storage to meet the metabolic demands of reproduction in later life (i.e., metabolic thrift). In this regarding, the same PCOS risk genes expressed in women with PCOS from both Chinese and European populations suggest ancient origins [68], potentially dating back before human diaspora out of sub-Saharan Africa 100,000–50,000 years ago [69, 70].

Indeed, commonality among > 20 PCOS candidate genes in women with differing PCOS phenotypes, including those diagnosed by Rotterdam or NIH criteria, or by self-report, strongly suggests shared molecular and developmental origins despite heterogeneity of PCOS phenotypic expression [71]. Moreover, rare gene variants of *DENND1A* involved in regulating androgen production, have been identified in ~ 50% of families with PCOS [72]. In this regard, a posttranscription form of *DENND1A*, DENND1A.v2, is over-expressed in some women with PCOS [73], while experimentally-induced DENND1A.v2 over-expression in human theca cells increases androgen biosynthesis and release as a fundamental PCOS trait linked to metabolic dysfunction. But without evidence of GWAS-identified, PCOS-associated, intronic gene variants of DENND1A enabling DENND1a.v2 over-expression or ovarian hyperandrogenism, investigations continue into the pathogenic mechanisms underlying this PCOS-associated gene variant [74].

A separate whole-genome sequencing study found in ~ 3% of families with PCOS, rare gene variants in anti-mullerian hormone (AMH) and its type 2 receptor (AMHR2) [75]. Both genes are involved in intra-ovarian follicle development and hypothalamic GnRH stimulation, which ultimately regulate ovarian androgen production [76]. Whether these mostly missense variants in AMH and AMHR2 induce the elevated circulating and intrafollicular levels of AMH and the ovarian hyperandrogenism of PCOS women remains unclear [75].

In addition, some PCOS candidate genes, such as thyroid adenoma associated (*THADA*) and insulin receptor (*INSR)*, have been associated with metabolic syndrome and impaired glucose regulation in PCOS and type 2 diabetes [77], suggesting genetic contributions to metabolic dysfunction in women with PCOS. Moreover, in a recent Mendelian randomization study, gene variants associated with high bioavailable (unbound) circulating T levels were also linked with PCOS and type 2 diabetes [78], providing an additional causal role for female hyperandrogenism in the onset of both disorders.

Maternal-fetal endocrine-metabolic dysfunction appears to enable genetic and/or epigenetic re-programming of the female phenotype into PCOS [79]. During the second trimester of development, the human fetal ovary contains several steroidogenic enzymes, genes encoding steroid-signalling pathways, and receptors to steroids, insulin and insulin-like growth factors, when primordial ovarian follicles and abdominal fat are present [1, 80, 81]. Mid-trimester human and nonhuman primate fetal ovaries can metabolize progestins and their conjugates into androgens enabling T secretion [82, 83]. The human midgestational fetal ovary also may produce androgens in response to in utero hyperinsulinemia, particularly in a female fetus with a genetic susceptibility to PCOS. Amniotic fluid T levels are elevated in female fetuses of diabetic [84] mothers, along with theca and pancreatic beta cell hyperplasia accompanying ovarian theca-lutein cysts in hirsute female stillbirth offspring of these women [85, 86]. Amniotic fluid T levels also are elevated in female fetuses of PCOS mothers [87] during mid-gestation when regional fat depots in the human fetus develop [81]. Elongated anogenital distances, as postnatal biomarkers of mid-gestational fetal hyperandrogenism, occur in both female infants of PCOS mothers and in PCOS women [5], affirming midgestational fetal female hyperandrogenism. Interestingly, term umbilical cord T levels are elevated in only some female infants of PCOS mothers since sex differences in fetal T exposure are normally minimal at birth [4]. These findings collectively suggest that endocrine-metabolic disorders of pregnancy in mothers with PCOS can induce epigenetic modifications of fetal genetic susceptibility to PCOS after birth.

From a maternal perspective, PCOS women in pregnancy have greater serum androgen levels, higher fasting and 2-h post-prandial insulin values and elevated serum AMH levels [88–90] compared to normal mothers. Subsequently, the prevalence of gestational diabetes, glucose intolerance and type 2 diabetes in PCOS women is up to 5-fold higher than that of other women and is worsened by obesity, with about 40% of PCOS women developing gestational diabetes and other pregnancy complications [5, 8]. Moreover, PCOS women in pregnancy exhibit exaggerated dyslipidemia and elevated circulating inflammatory markers that predict gestational diabetes, hypertensive disorders and adverse obstetrical/neonatal outcomes [91, 92]. Maternal hyperandrogenemia from PCOS, however, may not directly program PCOS in offspring if placental aromatization is normal [93]. Instead, metabolic dysfunction in a PCOS mother may be transmitted through the placenta to a female fetus with a genetic susceptibility to PCOS, promoting fetal hyperinsulinemia as a cause for hyperandrogenism and altered folliculogenesis in utero [5, 87, 94–96].

Alternatively, hyperandrogenic PCOS mothers [89] with elevated serum AMH levels in pregnancy [90] may have reduced placental aromatase expression [97], providing a potential maternal androgen contribution to female fetal hyperandrogenism. Evidence for this second mechanism is that gestational exposure of mice to recombinant AMH during a critical gestational age induces maternal neuroendocrine-driven hyperandrogenism and diminishes placental aromatization, causing a PCOS-like phenotype in female offspring and their descendants over multiple generations [90, 98].

### Animal models affirming developmental programming of PCOS through maternal-fetal endocrine-metabolic dysfunction

Gestational exposure of female nonhuman primates, sheep, rats, and mice to excess T, or dihydrotestosterone (DHT), induces reproductive and metabolic PCOS-like phenotypes resembling those of women with PCOS [99]. Such PCOS animal models provide unique perspectives on how hyperandrogenism and increased adiposity interact to affect PCOS phenotypic expression, given the worldwide human obesity epidemic [67].

As precocial species, in which tissue differentiation is completed during fetal life as in humans [100], prenatally T-treated monkeys and sheep provide particularly valuable mechanistic links between endocrine-metabolic dysfunction in pregnancy and its long-term metabolic-reproductive consequences in offspring. In prenatally T-treated monkeys, maternal glucose intolerance causes transient hyperinsulinemia in their female fetuses. Specifically, prenatal T-treatment in rhesus monkeys impairs maternal glucose tolerance and stimulates fetal insulin release, which then potentiates insulin action within the fetus [101]. Prenatal T-treatment in sheep also induces maternal hyperinsulinemia [102]. When these sheep are co-treated with either flutamide or rosiglitazone, juvenile insulin resistance and early adult hyperleptinemia are prevented [58].

Mid-gestational prenatal T-treatment in rhesus monkeys and sheep also programs adipose dysfunction with insulin resistance in adult offspring [94, 103]. Adult prenatally T-treated sheep with insulin resistance [58, 103, 104] also develop hypertension and hypercholesterolemia after puberty [105], although their long-term risks of developing increased adiposity and diabetes with age remain unclear. Prenatally-T treated juvenile female sheep also show increased stem cell commitment to preadipocytes and decreased preadipocyte differentiation of visceral adipocytes, with the latter prevented by dual prenatal flutamide/rosiglitazone co-treatment [57]. This increase in commitment may underlie the increased proportion of small adipocytes observed in both prenatal T-treated sheep and monkeys [55, 56].

Naturally-occurring female hyperandrogenism also occurs in some adult female macaques accompanied by PCOS-like endocrine-metabolic traits [106, 107]. A positive correlation of anogenital distance with circulating T levels in these adult monkeys suggests mid-gestational hyperandrogenic origins. Most naturally hyperandrogenic female macaques are not overweight [106], and resemble normal-weight women with PCOS [26], while those with the highest T values exhibit increased BMI, central adiposity and insulin resistance [107]. Since rhesus monkeys share > 97% DNA sequence homology with humans at protein-coding exons, evolutionary changes in the rhesus monkey exome that resemble those previously identified in human PCOS candidate genes could generate common biological phenotypes with initial selective advantages but comparable physiological consequences across primates [108].

### Clinical interventions that diminish metabolic dysfunction ameliorate PCOS symptomology

Potentially programmed in part during gestation, an altered metabolic phenotype in women with PCOS could predispose them to excess weight gain in today’s obesogenic environment, emphasizing the need for appropriate clinical strategies to improve their health, reduce their risks of developing maternal-fetal complications and optimize the long-term health of their offspring [51]. Although weight loss in overweight/obese women with PCOS through lifestyle intervention, medication use and/or bariatric surgery can improve their metabolic-reproductive function [5, 109, 110], long-term effects of these clinical therapies remains uncertain, while gestational use of some medications are either contraindicated (i.e., antiandrogens) or associated with childhood adiposity and insulin resistance (i.e., metformin) [111]. A more effective strategy may be to identify young girls at risk for PCOS, perhaps by measuring facial sebum content [112], anogenital distance [113] and/or circulating AMH levels [114] in early life, and then initiate relevant interventions before puberty. Such a clinical strategy for the treatment of PCOS shifts the paradigm from disease treatment to preventive intervention, stressing early and appropriate lifestyle choices and the development of novel therapies to improve the fertility and endocrine-metabolic health of PCOS women, reduce their risks of maternal-fetal complications and optimize the long-term health of their offspring.

## Conclusions

**Fig. 2:**
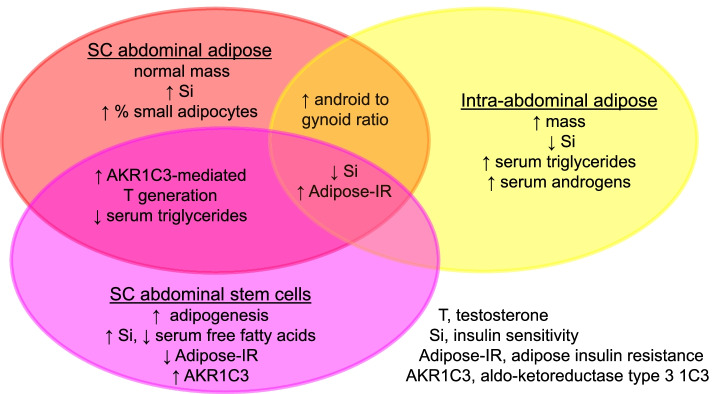
Metabolic adaptation in polycystic ovary syndrome. Inherently accelerated adipogenesis, along with enhanced intracellular aldo-ketoreductase type 1C3-mediated testosterone generation, in subcutaneous (SC) abdominal adipose promotes lipid storage (through increased lipogenesis and decreased lipolysis) to protect against insulin resistance. Simultaneously, hyperandrogenemia accompanies preferential accumulation of highly-lipolytic intra-abdominal fat with an opposite effect. As a result, SC fat storage counterbalances increased circulating glucose and free fatty acid (FFA) availability for energy use. When energy intake exceeds fat storage capacity, excess fatty acid influx into skeletal muscle and liver promotes lipotoxicity through ectopic lipid accumulation accompanied by oxidative stress, insulin resistance and inflammation in non-adipose tissue

Polycystic ovary syndrome has persisted from antiquity to become the most common reproductive-metabolic disorder of reproductive-aged women. Its ancestral traits once favored abdominal fat deposition and increased energy availability through hyperandrogenism and insulin resistance, respectively, for reproduction during food deprivation. These same traits in today’s environment, however, underlie different PCOS phenotypes with variable risks for subfertility and metabolic dysfunction that are worsened by obesity. Recent studies of healthy normal-weight women with NIH-defined PCOS show enhanced AKR1C3 activity in SC abdominal adipose favoring lipid storage in combination with preferential intra-abdominal fat deposition accompanying hyperandrogenemia and low-normal insulin sensitivity. Potentially programmed as an ancestral trait by genetic inheritance and epigenetic events during early life, such a metabolic adaptation in these normal-weight PCOS women provides a balance between enhanced SC adipose TG storage and increased circulating glucose and FFA availability as energy substrate for crucial target tissues, including brain and muscle (Fig. 2). It also favors subfertility from infrequent ovulation, perhaps allowing women from antiquity sufficient time and strength for childrearing of fewer offspring who have an enhanced likelihood of childhood survival [67]. Future studies should examine how heritable PCOS characteristics are influenced by today’s obesogenic environment through an epigenetic-related metabolic adaptation that favors fat storage, but predisposes to lipotoxicity with excess weight gain and pregnancy complications. Such investigations should focus on a new perspective that PCOS may have evolutionary origins in both human and nonhuman primates.

## Data Availability

Not applicable.

## Abbreviations

PCOS: Polycystic ovary syndrome
PCOM: Polycystic ovarian morphology
GWAS: Genome-wide association studies
BMI: Body mass index
Si: Insulin sensitivity
FSIVGTT: Frequently sampled intravenous glucose tolerance testing
HOMA-IR: Homeostatic Model Assessment for Insulin Resistance
adipose-IR: Adipose insulin resistance
TG: Triglyceride
non-HDL: Non-high density lipoprotein
AKR1C3: Aldo-ketoreductase type 1C3
AKR1C2: Aldo-ketoreductase type 1C2
T: Testosterone
A4: Androstenedione
SC: Subcutaneous
ASCs: Adipose stem cells
ZFP423: Zinc-finger protein 423
NIH: National Institutes of Health
PKA-RegIIβ: Protein kinase A regulatory-IIβ component
GLUT-4: Glucose transporter type 4
HSL: Hormone-sensitive lipase
*PPARγ*: Peroxisome proliferator-activated receptor gamma
*CEBPa*: CCAAT enhancer binding protein alpha
AP-1: Activator protein-1
FFA: Free fatty acid
AMH: Anti-mullerian hormone
LH: Luteinizing hormone
SHBG: Sex hormone binding globulin
TGF: Transforming growth factor
AMHR2: AMH type 2 receptor
*THADA*: Thyroid adenoma associated
*INSR*: Insulin receptor
DHT: Dihydrotestosterone
